# Who plays a more crucial role in adolescent well‐being: Interactions with parents or peers? An investigation of adolescents aged 10 to 18 years

**DOI:** 10.1111/aphw.70128

**Published:** 2026-03-06

**Authors:** Ruyi Ding, Ang Xia, Junhao Pan, Peng Zhang, Tuo Liu

**Affiliations:** ^1^ Department of Psychology Sun Yat‐Sen University Guangzhou China; ^2^ Positive Psychology Research Center, Department of Psychology and Cognitive Science Tsinghua University Beijing China; ^3^ Institute of Psychology Goethe‐Universität Frankfurt am Main Frankfurt am Main Germany

**Keywords:** adolescent, local structural equation modeling, parent, peer, random intercepts cross‐lagged panel model, well‐being

## Abstract

Adolescence is a critical period during which interactions with parents and peers play key roles in shaping mental well‐being, yet there is ongoing debate regarding their relative importance. To address this gap, this research examines the relationships among communication with parents, social interactions with peers, and adolescents' mental well‐being using data from 33,824 Chinese adolescents (ages 10–18 years, *M*_age_ = 13.55, 47.43% female) across three data collection waves. A random intercepts cross‐lagged panel model (RI‐CLPM) was combined with local structural equation modeling (LSEM) to disentangle between‐person and within‐person effects and examine potential age‐related moderation. The results revealed significant correlations among the random intercepts of the three variables. The within‐person cross‐lagged effects of communication with parents on adolescents' mental well‐being were significant, but not vice versa. The within‐person cross‐lagged effects of mental well‐being on social interactions with friends were significant but not vice versa. Age moderated between‐person level association, with mental well‐being and peer interactions showing a U‐shaped trend and the association between mental well‐being and parent–adolescent communication increasing linearly across age. In conclusion, the findings suggest that parent–adolescent communication is important in predicting adolescents' mental well‐being and supporting positive peer interactions across adolescent development.

Adolescence, marked by hormonal and biological changes, is a time of profound psychological and social transformation; this makes adolescents vulnerable to mental health challenges (Orben et al., 2020; Rapee et al., 2019). Parents and peers, as two major socialization agents for adolescents, are crucial in shaping their psychological well‐being (Raja et al., 1992). However, findings regarding the relative contributions of family versus peer support to adolescent emotional functioning are mixed (Oldfield et al., 2016), with some studies highlighting the more important roles of parents (Formoso et al., 2000), and others emphasizing peers (Laible et al., 2000). These inconsistencies may reflect developmental differences, as the influence of parents and peers likely shifts across adolescent age (Liu et al., 2024; Steinberg & Monahan, 2007). Moreover, longitudinal studies examining parent and peer interactions and adolescent well‐being are limited, and few have separated stable individual differences from within‐person fluctuations, limiting our understanding of their dynamic contributions.

To address these gaps, this study analyzed data from a large sample of adolescents aged 10–18 years across three waves, with 3‐month intervals between each wave, spanning 31 provinces and 138 cities in China, to examine the reciprocal relationships between parental and peer interactions and adolescent well‐being. The random intercepts cross‐lagged panel model (RI‐CLPM) was used to separate trait‐like effects from within‐individual fluctuations, allowing for inferences about potential causal effects at the within‐individual level (Hamaker et al., 2015; Mulder & Hamaker, 2021). Moreover, to explore how the relative roles of parents and peers in adolescent well‐being shift across different developmental stages of adolescence, local structural equation modeling (LSEM) (Hildebrandt et al., 2016) was employed to investigate potential age‐related differences.

## MENTAL WELL‐BEING OF ADOLESCENTS

More than the absence of mental illness, mental well‐being encompasses both hedonic (positive feelings, affect, and emotions) and eudaimonic (positive functioning, mindset, and relationships) dimensions (Gautam et al., 2024; Koushede et al., 2019). The World Health Organization (2022) states that mental health is “a state of mental well‐being that enables people to cope with the stresses of life, realize their abilities, learn well and work well, and contribute to their community.” (WHO, 2022). Warwick–Edinburgh Mental Well‐being Scale (WEMWBS), for instance, is a widely used scale that assesses two major facets of positive mental health, namely, the hedonic perspective that focuses on the subjective experience of happiness and life satisfaction and the eudaimonic perspective that focuses on psychological functioning and self‐realization (Stewart‐Brown et al., 2009; Tennant et al., 2007). The scale has demonstrated good cross‐cultural validity, including in Chinese adolescent populations (Fung et al., 2025).

High levels of mental well‐being are important during adolescence when individuals are undergoing rapid changes that may increase their vulnerability to psychological distress and crises (Ekinci, 2024; Orben et al., 2020). For instance, adolescent mental well‐being influences their social and emotional development, shaping their ability to form relationships, manage stress, and build resilience (Ekinci, 2024). Robust mental well‐being serves as a protective factor against self‐harm thoughts and behaviors among youth (Russell et al., 2020). Adolescent well‐being is shaped by a complex set of international factors, such as physical health, self‐esteem, lifestyle choices, and mindfulness, as well as external factors, including environmental conditions, economic stability, education, leisure, safety, and social connections (Avedissian & Alayan, 2021; Rozi et al., 2023). Research into the protective and risk factors affecting adolescent mental health is critical, as it provides valuable insights for parents, educators, policymakers, and mental health professionals to create environments that enhance adolescents' ability to cope with challenges, thereby laying a strong foundation for their adult well‐being.

## THE COMPLEMENTARY ROLES OF SOCIAL INTERACTIONS IN ADOLESCENT WELL‐BEING

Self‐determination theory emphasizes that relatedness, referring to the need to feel connected to and valued by others, is an essential psychological need for fostering optimal human development, motivation, and well‐being (Baumeister & Leary, 1995; Deci & Ryan, 2012). During adolescence, parents and peers are two primary sources of social interaction, each playing a crucial role in shaping adolescents' well‐being (McMahon et al., 2020). The social reorientation hypothesis proposes that adolescents place increased reliance on peers for social and emotional support as they negotiate greater independence from their family and establish their “autonomous self” (Nelson et al., 2016; Nowell et al., 2023). With increasing individuation, adolescents become more engaged with their peers (Lam et al., 2014; Rubin et al., 2007). Difficulties in peer relationships, such as peer rejection, bullying, and loneliness, are risk factors for developing affective disorders such as depression during adolescence; conversely, high‐quality peer relationships can protect against mental health issues and enhance resilience in adolescents (Orben et al., 2020).

At the same time, despite communication with parents often becoming more challenging for both parents and adolescents (Noller & Bagi, 1985), adolescents who maintain a positive relationship with their parents are more likely to engage in meaningful communication, sharing their daily activities and disclosing their thoughts and feelings (Deković et al., 2004). Thus, the frequency of communication with parents is typically regarded as an important indicator of the quality of the parent‐adolescent relationship, providing insights into the openness and connectedness within the relationship. For example, adolescent disclosure to parents is positively associated with various aspects of adolescent adaptability, including better mental health and well‐being (Kerr & Stattin, 2000; Nowell et al., 2023; Stattin & Kerr, 2000). Collectively, these findings underscore that both parents and peers exert significant influences on adolescents' psychological well‐being. Supporting this, one study using polynomial regression and response surface analysis found that the self‐esteem of adolescents (aged from 10 to 24 years) was highest when attachment to both parents and peers was strong rather than low (Karunarathne, 2023).

## THE COMPETITIVE ROLES OF SOCIAL INTERACTIONS IN ADOLESCENT WELL‐BEING

Researchers are also interested in the relative importance of parents' and peers' roles in influencing adolescents' psychological well‐being, although different studies have reported inconsistent findings (Oldfield et al., 2016; Weinstein et al., 2006). According to attachment theory (Bowlby, 1969), parents may play a particularly important role, as secure parent–child relationships provide a “safe base” that supports adolescents' emotional regulation, psychological well‐being, and resilience. This perspective underscores that as children enter adolescence, the quality of parent–child interactions continues to shape internalized relationship models, guiding how adolescents engage with peers and navigate social challenges (Benson et al., 2006) and support adolescents' psychological development. For example, one study has shown that attachment to parents is more significant than attachment to peers in developing adolescents' self‐esteem for adolescents aged from 10 to 24 years (Karunarathne, 2023). Similarly, using the daily diary method, researchers have documented that the overall link between family support and adolescent mood was higher than the link between peer support and adolescent mood for middle and high school adolescents (Weinstein et al., 2006).

However, from the perspective of social interaction quantity, some researchers argue that peers may exert a stronger influence on adolescents' well‐being, as adolescents increasingly spend more time engaging with peers than with parents (Farley & Kim‐Spoon, 2014). Supporting this viewpoint, one study with adolescents (mean age = 16.1 years) has found that although both parent and peer attachment positively relate to adolescent adjustment, peer attachment appeared more influential, with adolescents high in peer but low in parent attachment showing better adjustment than those high in parent but low in peer attachment (Laible et al., 2000). Also, one meta‐analysis has shown that a small positive correlation exists between parent–child relationship quality and empathy, and a small‐to‐moderate positive correlation exists between peer relationship quality and empathy, which was significantly stronger than the correlation with parent–child relationship quality (Boele et al., 2019).

## THE NECESSARY TO DISTINGUISH WITHIN‐PERSON EFFECTS FROM BETWEEN‐PERSON EFFECTS

One possible explanation for the inconsistency of parents' versus peers' relative roles in adolescent adjustment may lie in the methods of past studies. Although some research has used longitudinal designs, many of these studies have not distinguished between within‐person changes and between‐person differences. Within‐person changes refer to how an individual adolescent's well‐being varies over time in response to changes in parental or peer interactions, providing stronger evidence of causal effects; in contrast, between‐person differences reflect how an adolescent who consistently receives more or less social interaction differs from one another in well‐being, which can indicate correlations but cannot establish causation (Hamaker et al., 2015; Mulder & Hamaker, 2021). Cross‐lagged panel models (CLPM), for example, can examine longitudinal relationships between variables, but they cannot distinguish between stable differences across individuals (between‐person effects) and changes within the same individual over time (within‐person effects) (Hamaker et al., 2015; Mulder & Hamaker, 2021). Recent researchers have advocated for the use of the Random‐Intercept CLPM (RI‐CLPM) in longitudinal studies, as it effectively distinguishes within‐person changes over time from stable between‐person differences (Hamaker et al., 2015; Mulder & Hamaker, 2021). Thus, this study would employ a longitudinal design using RI‐CLPM to capture the dynamic and reciprocal relationship between parental and peer interactions on adolescent well‐being.

## THE MODERATING ROLES OF AGE

The inconsistencies concerning the relative importance of parents or peers may also reflect developmental differences across early (around 10 to 13 years), middle (around 14 to 17 years), and late adolescence (around 18 to 21+ years). Overall, past literature indicates that parental influence on adolescents' development tends to decline and peer influence tends to increase from early to middle adolescence. For example, a U‐shaped pattern has been observed in the relationship between parental depression and adolescent internalizing problems across age, with a strong initial correlation in early adolescence, followed by a decrease in middle adolescence, and then a subsequent increase in late adolescence (Liu et al., 2024). Also, early and middle adolescents reported placing more value on being in a popular group and perceived more group conformity and leadership within their groups than pre‐adolescents and late adolescents (Gavin & Furman, 1989). Similarly, some researchers believe that the development of susceptibility to peer pressure in adolescence follows an inverted U‐shaped curve, increasing during early adolescence, peaking around age 14, and declining thereafter (Steinberg & Monahan, 2007). Thus, from early to middle adolescence—roughly spanning late primary school to middle school and into high school, the relationships between parent‐adolescent/peer interaction and adolescents' well‐being may be moderated by age. Further empirical research is needed to elucidate the specific ways in which age moderates the relationships between parent–adolescent and peer interactions and adolescents' well‐being.

To better understand how developmental factors such as age moderate the relationships between social interactions and adolescent mental well‐being, we propose using LSEM (Hildebrandt et al., 2016). Unlike the typically used multigroup SEM approach or moderated nonlinear factor analysis (Kush et al., 2023), LSEM allows the examination of how relationships between variables may nonlinearly and continuously change across the continuum of a moderator, without requiring categorization or specific nonlinear assumptions (Liu et al., 2024). Importantly, although RI‐CLPM is well‐established for distinguishing stable between‐person differences from dynamic within‐person changes, and LSEM is recognized for its flexibility in capturing continuous and nonlinear moderating effects, their integration has not been explored in previous research. This research seeks to advance methodological approaches, providing a more nuanced analysis of variable relationships across the adolescent developmental trajectory by integrating LSEM into the RI‐CLPM framework. We believe that this integration can offer a comprehensive understanding of how between‐person and within‐person effects evolve in the context of developmental moderators (i.e., age), ultimately facilitating more targeted and effective interventions.

## THE PREDICTING ROLES OF YOUTH WELL‐BEING ON SOCIAL INTERACTION

Furthermore, researchers have emphasized the importance of considering the bidirectional influence between youth well‐being and relationship context (Farley & Kim‐Spoon, 2014; Lerner & Castellino, 2002). This perspective suggests that interactions with parents and peers impact adolescent well‐being and that adolescent well‐being reciprocally influences these interactions. Although this bidirectional process is theoretically plausible, most existing research relies on cross‐sectional designs, which provide only a static snapshot and limit the understanding of these dynamic interactions. Empirical evidence, however, supports the possibility of such reciprocal effects. For example, adolescents with initially stronger emotional repair abilities—to actively change negative moods to more positive moods—were rated as increasingly socially competent over time, and both displayed and experienced increasingly positive interactive behaviors with close peers over time (Hessel et al., 2016). Lower levels of self‐esteem at age 15 years were associated with lower levels of life and relationship satisfaction at ages 18, 21, and 25 years (Boden et al., 2008). One study using a cross‐lagged analysis has supported that the bidirectional relationship exists between Chinese adolescents' depression and parental rejection and peer attachment over time (Yu et al., 2024). However, as noted earlier, traditional CLPMs cannot separate within‐ and between‐person effects, highlighting the need for longitudinal approaches, such as RI‐CLPM, to disentangle the reciprocal interplay between adolescents' well‐being and their relationships with parents and peers.

## A FOCUS ON CONTEMPORARY CHINESE ADOLESCENTS

Of note, the above‐mentioned findings regarding the relationships between parent and peer interactions and adolescent development are primarily based on Western samples. The roles of parents and peers in adolescent well‐being merit investigation in China because cultural values may shape these influences differently than in Western populations. In Chinese culture, greater emphasis is placed on relatedness rather than autonomy, meaning that family ties and parent–child relationships may remain particularly central compared with peer interactions. For example, research has found that in Asian cultural contexts, parental control may not be perceived by adolescents as less intrusive to their sense of self compared with European American cultures (Wang et al., 2007). The beneficial effects of parents' psychological autonomy support were generally stronger in the United States than in China (Wang et al., 2007). Also, Chinese (vs. American) children appear to be less concerned with individuating from parents during early adolescence (Pomerantz et al., 2009; Xiong et al., 2020). Thus, in contemporary Chinese adolescents, cultural emphasis on family‐relatedness and parental authority may shape the roles of parents and peers in adolescent adjustment, underscoring the need to examine both influences within the Chinese context.

## THE PRESENT RESEARCH

The current research aimed to investigate the longitudinal relationships between social interactions with parents and peers and adolescent well‐being, using a three‐wave design with assessments spaced 3 months apart. We examined communication frequency with parents and social interaction frequency with peers. Communication frequency with parents focuses on how often adolescents engage in conversations and connect with their parents both in person and online (Rudi et al., 2015). Communication with parents is considered a key indicator of a healthy parent‐adolescent relationship and is crucial in supporting a successful transition into adulthood. Communication provides opportunities for parents to know about adolescents' thoughts and behaviors and fosters understanding, trust, and emotional closeness, which are essential for adolescents' adjustment (Ding et al., 2022; Keijsers & Poulin, 2013; Miller‐Day, 2002). Social interaction frequency with peers encompasses the time spent and activities shared with friends both in person and online, highlighting the role of peer contact (Ralph et al., 1995; Twenge et al., 2019).

Our objective is to explore the bidirectional influence between youth well‐being and relationship context using RI‐CLPM, which distinguishes within‐person changes from stable between‐person differences (Hamaker et al., 2015; Mulder & Hamaker, 2021). The second objective was to examine the moderating effect of age on the relationships between social interaction and adolescent mental well‐being. We included a sample ranging from 10 to 18 years old, covering the key stages of adolescence.

Four groups of hypotheses were made. First, we hypothesized that mental well‐being, communication with parents, and social interaction with peers would be stable over 6 months. Thus, within‐person autoregressive effects for mental well‐being, communication with parents, and social interaction with peers would be significant across consecutive time points (Hypothesis 1). Second, we expected that increased interactions with parents and peers would predict higher adolescent well‐being, both at the between‐person (Hypothesis 2a) and within‐person (Hypothesis 2b) levels. When analyzing both parent‐adolescent and peer interaction, the former may be more likely to predict adolescent well‐being (Hypothesis 2c). Third, we anticipated that higher adolescent well‐being would predict more interactions with parents and peers over time, again at both the between‐person (Hypothesis 3a) and within‐person (Hypothesis 3b) levels. Fourth, we anticipated age‐related moderating effects in the relationship between adolescent well‐being and their interactions with parents and peers (Hypothesis 4). Given that the moderating effect could be linear or nonlinear, we refrained from hypothesizing about its form. Lastly, we hypothesized that parent‐adolescent communication would predict the frequency of social interactions with peers at the within‐person level, but the reverse relationship would not hold (Hypothesis 5a). However, for between‐person effects, we expected the two variables to be correlated (Hypothesis 5b).

## METHODS

### Participants and procedure

We used data from a longitudinal research project investigating environmental factors and adolescent development in China, involving adolescents aged from 10 to 18 years across 31 provinces and 138 cities in China (Zhang et al., 2023). The adolescents completed online surveys in three waves, spaced 3 months apart. In Wave 1, 417,144 participants were surveyed; 39,820 responses were deemed valid and became the focus of the study (Zhang et al., 2023). Of those, 35,079 and 33,824 participated in Waves 2 and 3, respectively. Thus, 33,824 participants completed all three waves and became the primary focus of this study. Their average age was 13.55 years (*SD* = 2.64), 47.43% were females, 77.94% were from urban areas, and 79.39% were from public schools. Participant demographics are shown in Table S1.

The online survey was created using “Wenjuanxing,” a widely used platform in China. The students were recruited through schools. Questionnaires were completed on computers by students in school settings under the supervision of their head or psychological teachers, who received 1 day of standardized training from the research team. Students' assent to participate and parental informed consent were obtained. The research was approved by the ethical board of the corresponding author's University.

### Measures

#### Mental well‐being

Adolescent mental well‐being was assessed using the short version of the Warwick–Edinburgh Mental Well‐Being Scale (SWEMWBS; Stewart‐Brown et al., 2009; Tennant et al., 2007). The scale includes seven items (e.g., “I've been feeling relaxed”), with each rated from 1 (*none of the time*) to 5 (*all of the time*). The scale has good reliability and validity in China (Fung, 2019). In this study, the reliability measured by the alpha and omega coefficients in each wave ranged from .91 to .95. Following past literature (Zhang et al., 2023), a composite score was computed by summing all the items, where higher scores indicated greater degrees of mental well‐being.

#### Frequency of communication with parents

The frequency of communication with parents was assessed using two items: (1) “In the past months, how often have you had face‐to‐face conversations with your parents?” and (2) “In the past months, how often have you communicated with your parents online (including phone calls, WeChat/QQ video or voice chats, etc.)?” Participants gave their responses from 1 to 6, where 1 = *only once or twice in total/almost never*, 2 = *once a month or less*, 3 = *two to three times a month*, 4 = *once or twice a week*, 5 = *three to four times a week*, and 6 = *nearly every day*. A composite score was computed by summing the two items, where higher scores indicated a greater frequency of communication with parents.

#### Social interactions with friends

Social interactions with friends were assessed using two items: (1) “How many days per week do you usually spend socializing with friends or classmates, whether it's meeting up, chatting, playing, or doing other activities together?” and (2) “How many days per week do you usually chat, play games, or do other activities with friends or classmates online (through the internet, video calls, WeChat, etc.)?” Participants responded from 0 to 7, where the number represents the number of days. A composite score was computed by summing the two items, where higher scores indicated a greater frequency of social interactions with friends.

### Data analysis

#### Invalid data detection

As a common method to detect insufficient response, Mahalanobis distances were calculated for adolescent mental well‐being in three waves (Meade & Craig, 2012). Mental well‐being was selected as the focal variable for outlier detection because it served as the primary outcome variable in our theoretical model, and extreme or inconsistent patterns in well‐being scores could indicate careless or random responding patterns that would violate the assumptions of our longitudinal modeling approach. The critical cutoff of the *χ* ^2^ distribution was used to determine the threshold, and the 0.975 quantile was used (equivalent to the 0.05 significance level of the two‐tailed test). The participants whose mental well‐being scores had Mahalanobis distances exceeding the cutoff in at least one wave were excluded from the analysis (Curran, 2016). The frequency of communication with parents and social interactions with friends was a direct summation and not latent construct; thus, using this method was not necessary for them.

#### Longitudinal measurement invariance analysis

Longitudinal measurement invariance analysis (Widaman et al., 2010) was conducted for SWEMWBS across the three time points. The scales measuring the frequency of communication with parents and social interactions with friends each consisted of only two items, rendering them unsuitable for testing measurement invariance.

The assessment of longitudinal measurement invariance was conducted through a hierarchical sequence: (1) configural invariance, establishing a baseline by asserting a time‐consistent one‐factor structure across the three points; (2) metric invariance, maintaining time‐consistent factor loadings (*λ*) while allowing item intercepts (*τ*) and residual variances (*ε*) to vary across time points; (3) scalar invariance, upholding time‐consistent factor loadings (*λ*) and item intercepts (*τ*), while permitting residual variances (*ε*) to fluctuate across time points. Model fit was evaluated using comparative fit index (CFI), the Tucker–Lewis index (TLI), root mean square error of approximation (RMSEA), and standardized root mean square residual (SRMR), with CFI > 0.90, TLI > 0.90, RMSEA < 0.08, and SRMR < 0.08 indicating acceptable fit (Hu & Bentler, 1999). At each stage, measurement invariance is met if the changes in fit indices between models are within acceptable thresholds: ΔCFI ≤ −0.010, ΔTLI ≤ −0.010, ΔRMSEA ≤ +0.015, and ΔSRMR ≤ +0.030 for loading invariance and ≤0.010 for intercept invariance (Chen, 2007).

#### Means, standard deviations, and correlations

Means, standard deviations, and bivariate correlations for all measures were computed using R 4.4.2.

#### RI‐CLPM

To investigate the bidirectional relations among the three assessed variables, we employed RI‐CLPM (Hamaker et al., 2015) using the *lavaan* package in R (Version 4.4.2; Rosseel, 2012). The RI‐CLPM can differentiate effects on the within‐person level (i.e., autoregressive paths, concurrent paths, and cross‐lagged paths) from effects on the between‐person level (i.e., random intercepts; Usami, 2021).

Following past literature (Mulder & Hamaker, 2021), we tested four models by systematically adding constraints: (1) unrestricted model (Model A), without constraints, allowing all parameters to vary freely; (2) autoregressive equality model (Model B), with equality constraints on autoregressive effects across time points; (3) cross‐lagged equality model (Model C), with equality constraints on cross‐lagged effects across time points; (4) autoregressive and cross‐lagged equality model (Model D), with equality constraints on both autoregressive and cross‐lagged effects. Model fit was considered acceptable if CFI > 0.90, RMSEA < 0.08, and SRMR < 0.08 (Hu & Bentler, 1999). To compare the constrained and unconstrained models, the *χ* ^2^ difference test was conducted. In all analyses, missing data were handled using full information maximum likelihood estimation.

#### Sensitivity analysis of RI‐CLPM

To assess whether the exclusion decision affected our substantive conclusions, we conducted a sensitivity analysis by re‐estimating the primary RI‐CLPM model using the complete dataset, including all 33,824 participants.

#### LSEM

We used LSEM (Hildebrandt et al., 2016; Liu et al., 2024) to examine the moderating effects of student age. The *lsem.estimate()* function implemented in *sirt* R‐package (Robitzsch, 2024) was used. For a comprehensive guide on implementing LSEM using the R‐*sirt* package, please refer to Liu et al. (2024). To determine the appropriate sample size for each age group, we conducted a power analysis using the function *powRICLPM()* (Mulder, 2023). The analysis was based on three measurement occasions with three primary variables. We assumed moderate autoregressive effects (*β* = .30) and small‐to‐moderate cross‐lagged effects (*β* = .20), with intraclass correlation coefficients of .50 for all variables. Power analysis indicated that a minimum sample size of 1411 participants would be required to achieve 80% power (*α* = .05) for detecting the hypothesized cross‐lagged effects. For LSEM, we moderated the RI‐CLPM across student ages ranging from 10 to 17. However, values at the borders of the distribution (8, 9, 18, 19, and 20) were categorized into adjacent age groups (e.g., Age Groups 8 and 9 were categorized into Age Group 10), because the effective sample size was low for these moderator values. The histogram of age is shown in Figure S1. Based on Hildebrandt et al. (2016), we used a bandwidth parameter of 2.

##### Preliminary examination of the moderating effect of age

To obtain the significance of the potential moderating effect of age, parametric bootstrapping was first used to explore the moderating effect of age on a particular parameter by function *lsem.bootstrap()*. We used a 1000‐sample parametric bootstrapping, obtaining the mean (*M*) and the standard deviation (*SD*) of the parameter of interest in SEM across moderator values (Hildebrandt et al., 2016; Olaru et al., 2020). Using the *t*‐test formula of parameter variance, the *p*‐value for *SD* indicates whether parameters change significantly across different moderator values. Second, we implemented a permutation test using the *lsem.permutationTest()* function to obtain an additional *p*‐value for the *SD*. The moderating effect was considered significant only when both approaches yielded significant *p*‐values for the SD, indicating robust evidence that parameters systematically varied across different age values.

##### In‐depth analysis of the moderation effect of age: Linear versus nonlinear

When a significant moderation effect was detected for a specific parameter in SEM, we employed the *lsem.test()* function to determine whether a specified hypothesized parametric function could adequately describe the nonparametric, nonlinear moderation effect (Liu et al., 2024). In this study, we tested whether a linear (indicating linear change) and quadratic (indicating U‐shaped change) sufficed to characterize the age‐related moderation effect. Therefore, the following models were evaluated sequentially: (1) a linear model, including both the intercept and the linear regression coefficient, (2) a quadratic model, incorporating the intercept along with both linear and quadratic regression coefficients (allowing for U‐shaped or inverted U‐shaped curves), and (3) models incorporating the intercept along with both linear and even higher‐degree terms (cubic, quartic, and quintic) to capture more complex relationships. To assess the adequacy of each model, we applied the test of sufficient fit (*chisq_fit*) to assess whether the current model (e.g., linear or quadratic) adequately captured the nonparametric curve by comparing the hypothesized parametric function with the nonparametric local fit, which refers to a flexible, data‐driven method of fitting a curve or relationship between variables without assuming a specific parametric form. For the linear model, a nonsignificant result in the test of sufficient fit suggests that age influences the relationship linearly, whereas a significant result implies nonlinear age moderation effects. For the quadratic model, a nonsignificant result in the test of sufficient fit suggests quadratic nonlinear age moderation effects, whereas a significant result indicates that the quadratic model is inadequate, and a more complex model may be required. Finally, we reported the fit information with individual coefficients for the selected model—either the linear or quadratic model—that sufficiently described the moderating effects.

## RESULTS

### Invalid data detection

A total of 6297 participants whose mental well‐being scores had Mahalanobis distances exceeding the cutoff in at least one wave were excluded from the analysis. Thus, 27,527 participants were included in the analysis. Past literature suggests that invalid survey responses in studies can reach as high as 30% (Cornell et al., 2014). In this context, the present research exclusion rate of approximately 18.62% is well within a reasonable range, supporting the validity of the research methodology and data processing.

### Longitudinal measurement invariance analysis

Table 1 presents the longitudinal measurement invariance analyses for the SWEMWBS. Measurement equivalence was achieved at the strict level, indicating time‐invariance for the structure, factor loadings, and intercepts. Thus, adolescent mental well‐being was measured consistently across identical waves.

**TABLE 1:** Longitudinal measurement invariance of mental health symptom construct.

	*χ* ^2^ (*df*)	*p*	CFI	RMSEA [95% CI]	SRMR
Level of invariance across time‐points					
Configural (varying *λ*, *τ*, *ε*)	30541.546 (186)	< .001	.932	.077 [.076, .078]	.027
Metric (equal *λ*, varying *τ*, *ε*)	30735.296 (198)	< .001	.932	.075 [.074, .076]	.029
Scalar (equal *λ*, *τ*, varying *ε*)	32168.447 (210)	< .001	.929	.074 [.074, .075]	.033

	Δχ^2^ (*df*)	*p*	ΔCFI	ΔRMSEA	ΔSRMR
Tests of measurement invariance across time‐points					
Metric vs. configural	193.75 (12)	< .001	−.000	−.002	.002
Scalar vs. metric	1433.15 (12)	< .001	−.003	−.001	.004

*Note*: *λ* = item loading; *τ* = item intercept; *ε* = item error variance.

Abbreviations: CFI, comparative fit index; RMSEA, root mean square error of approximation; SRMR, square root mean residual.

### Means, standard deviations, and correlations

Means, standard deviations, and bivariate correlations for all measures are shown in Table 2.

**TABLE 2:** Means, standard deviations, and correlations.

No.	Variable	*M*	*SD*	1	2	3	4	5	6	7	8
1	Mental well‐being (Wave 1)	25.84	5.37								
2	Mental well‐being (Wave 2)	24.88	5.53	0.54							
3	Mental well‐being (Wave 3)	24.89	5.86	0.50	0.60						
4	Communication with parents (Wave 1)	9.11	2.55	0.24	0.20	0.16					
5	Communication with parents (Wave 2)	8.99	2.56	0.17	0.24	0.18	0.41				
6	Communication with parents (Wave 3)	9.05	2.56	0.15	0.17	0.19	0.37	0.45			
7	Social interactions with friends (Wave 1)	6.91	3.95	0.11	0.07	0.05	0.13	0.07	0.07		
8	Social interactions with friends (Wave 2)	6.81	3.97	0.06	0.11	0.05	0.07	0.11	0.06	0.36	
9	Social interactions with friends (Wave 3)	6.87	4.02	0.07	0.09	0.13	0.06	0.07	0.08	0.33	0.42

### RI‐CLPM

#### Model comparison

All models demonstrated satisfactory fitting results (Table 3). Considering *χ* ^2^ difference tests and these fitting indicators comprehensively, Model C, with constraints on the cross‐lagged effects, was deemed to be the best model.

**TABLE 3:** Results of the four models.

Model constraints	*χ* ^2^ (*df*)	Model	CFI	RMSEA [95% CI]	SRMR
Unrestricted model	167.52 (9)	a	0.997	0.025 [0.022, 0.029]	0.011
Autoregressive equality model	523.81 (12)	b	0.990	0.039 [0.037, 0.042]	0.018
Coupling effects set equal over time	195.00 (15)	c	0.996	0.021 [0.018, 0.024]	0.012
Autoregressive and coupling equality model	542.89 (18)	d	0.990	0.033 [0.030, 0.035]	0.019

	Δχ^2^ (*df*)	*p*	ΔCFI	ΔRMSEA	ΔSRMR
a vs. b	356.29 (3)	<.001	−0.007	0.014	0.007
a vs. c	27.49 (6)	<.001	−0.001	−0.004	0.001
c vs. d	347.89 (3)	<.001	−0.006	0.022	0.007

#### Between‐ and within‐person associations over time

**FIGURE 1:**
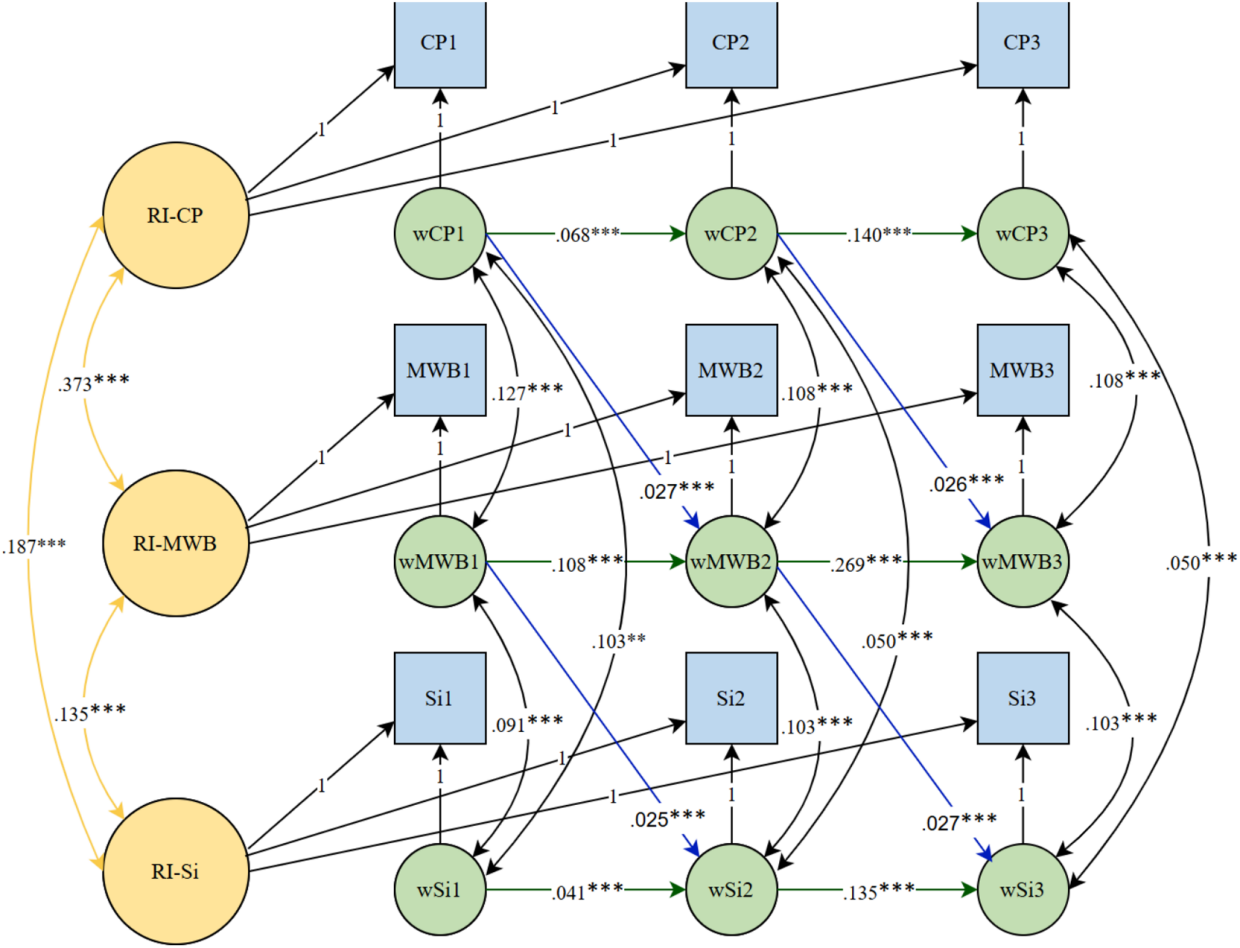
Using random‐intercept cross‐lagged panel model (RI‐CLPM) to estimate the relations between mental well‐being, frequency of communication with parents, and social interactions with friends. CP, communication with parents; MWB, mental well‐being; RI, random intercept; Si, social interactions with friends; w, within‐person; 1 = Wave 1; 2 = Wave 2; 3 = Wave 3. All autoregressive and cross‐lagged paths were estimated, but only social interactions with significant ones are presented. ***p < .001.

Table 4 presents the standardized estimates for random intercept correlations, within‐person autoregressive paths, and cross‐lagged effects in Model C, with the path diagram shown in Figure 1.

**TABLE 4:** Estimates of within‐person autoregressions and within‐person cross‐lagged effects in the random‐intercept cross‐lagged panel model (RI‐CLPM).

Estimation	T1 → T2		T2 → T3	
	β	p	β	p
Within‐person autoregressions				
Mental well‐being → mental well‐being	.108 [0.085, 0.131]	<.001	.269 [0.250, 0.288]	<.001
Communication with parents → communication with parents	.068 [0.049, 0.087]	<.001	.140 [0.121, 0.159]	<.001
Social interactions with friends → social interactions with friends	.041 [0.022, 0.060]	<.001	.135 [0.116, 0.154]	<.001
Within‐person cross‐lagged effects				
Communication with parents → mental well‐being	.027 [0.014, 0.040]	<.001	.026 [0.013, 0.039]	<.001
Social interactions with friends → mental well‐being	−.004 [−0.018, 0.010]	.550	−.004 [−0.018, 0.010]	.550
Mental well‐being → communication with parents	.012 [−0.001, 0.025]	.074	.014 [−0.002, 0.030]	.074
Mental well‐being → social interactions with friends	.025 [0.012, 0.038]	<.001	.027 [0.013, 0.041]	<.001
Communication with parents → social interactions with friends	−.002 [−0.016, 0.012]	.804	−.002 [−0.016, 0.012]	.804
Social interactions with friends → communication with parents	−.007 [−0.021, 0.007]	.319	−.007 [−0.021, 0.007]	.319

*Note*: The standardized estimates are shown. For the *χ* ^2^ test, the significance test should show *p* ≥ .05 (because the null hypothesis is that the model fits the data; this should not be rejected).

Abbreviations: CFI, comparative fit index; RMSEA, root mean square error of approximation (and 90% confidence intervals); TLI, Tucker–Lewis index.

As shown in Table 4, the within‐person autoregressive effects for mental well‐being, frequency of communication with parents, and social interactions with friends were statistically significant (*p*s < .001). This indicates that for the three assessed variables, when adolescents deviated from their mean score at one time point, deviation in the same direction was expected at the next occasion. Moreover, at the within‐person level, the cross‐lagged effect of frequency of communication with parents on mental well‐being (*p* < .001) was statistically significant, but the cross‐lagged effect of mental well‐being on the frequency of communication with parents was not significant (*p* > .05). This indicates that frequency of communication with parents significantly predicts future better mental well‐being but not vice versa. The cross‐lagged effect of social interactions with friends on mental well‐being (*p* > .05) was not statistically significant, but mental well‐being had a significant predicting effect on social interactions with friends (*p* < .001). This indicates that mental well‐being predicts more future social interactions with friends but not vice versa. Additionally, no significant cross‐lagged effects were found between social interactions with friends and the frequency of communication with parents (*p* > .05).

At the between‐person level, a significant correlation was found between the random intercepts of mental well‐being and frequency of communication with parents (*p* < .001, see Figure 1), meaning that adolescents with higher scores on mental well‐being scored higher on communication with parents and vice versa. Similarly, a significant correlation was observed between random intercepts of mental well‐being and social interactions with friends (*p* < .001, see Figure 1), meaning that adolescents with higher scores on mental well‐being scored higher on social interactions with friends and vice versa. Additionally, a significant correlation was found between the random intercepts of communication with parents and social interactions with friends (*p* < .001, see Figure 1), suggesting that deviations in both variables co‐occurred in the same direction.

#### Sensitivity analysis of RI‐CLPM

To evaluate the robustness of our findings to the outlier exclusion decision, we re‐estimated the RI‐CLPM model using the complete sample (*n* = 33,824, including the 6297 cases identified as outliers). Table S2 presents the results of the sensitivity analysis.

The sensitivity analysis demonstrated that our primary substantive conclusions were highly robust to the outlier exclusion decision. However, although the within‐person effect of adolescent well‐being on subsequent parent communication was marginally non‐significant in the primary analysis (*p* = .074), the association remained positive and became statistically significant in the sensitivity analysis (*p* = .007). Overall, the sensitivity analyses demonstrate that our core findings are not dependent on the outlier exclusion decision, with all significant effects showing consistent directions and similar magnitudes across both analyses.

### LSEM

**FIGURE 2:**
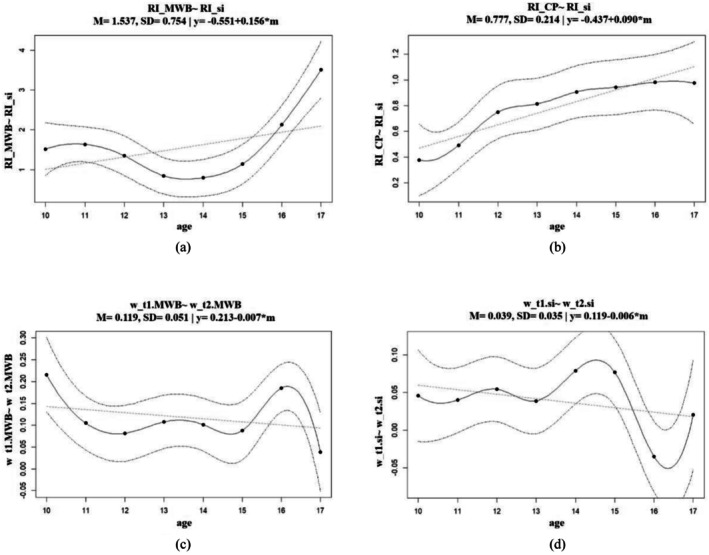
(a–d) Parameter curves of bootstrapping. CP, communication with parents; MWB, mental well‐being; RI, random intercept component; Si, social interactions with friends; w, within‐person component.

The bootstrapping and permutation test results revealed that the cross‐lagged effects did not exhibit a significant variation across different ages (*SD*_*ps* > .05, see Nos. 1–12 in Tables 5 and Figure 2a,b).

**TABLE 5:** Moderation analysis using local structure equation model.

No.	Effects	*M*	*SD*	Preliminary examination of age's moderation effect		In‐depth analysis of age's moderation effect *p*_fit of *chisq_het*	
				*SD_p* bootstrapping	*SD_p* permutation test	Linear vs. local model	Quadratic vs. local model
Within‐person cross‐lagged effects							
1	w_CP at wave (T1) → w_MW at wave (T2)	0.038	0.035	.151	.295		
2	w_CP at wave (T2) → w_MW at wave (T3)	0.038	0.035	.151	.295		
3	w_SIF at wave (T1) → w_MW at wave (T2)	0.004	0.017	.500	.592		
4	w_SIF at wave (T2) → w_MW at wave (T3)	0.004	0.017	.500	.592		
5	w_MW at wave (T1) → w_CP at wave (T2)	0.005	0.008	.500	.528		
6	w_MW at wave (T2) → w_CP at wave (T3)	0.005	0.008	.500	.528		
7	w_SIF at wave (T1) → w_CP at wave (T2)	−0.003	0.006	.500	.858		
8	w_SIF at wave (T2) → w_CP at wave (T3)	−0.003	0.006	.500	.858		
9	w_MW at wave (T1) → w_SIF at wave (T2)	0.024	0.014	.173	.345		
10	w_MW at wave (T2) → w_SIF at wave (T3)	0.024	0.014	.173	.345		
11	w_CP at wave (T1) → w_SIF at wave (T2)	0.002	0.028	.085	.231		
12	w_CP at wave (T2) → w_SIF at wave (T3)	0.002	0.028	.085	.231		
Within‐person autoregression effects							
13	w_MW at wave (T1) → w_MW at wave (T2)	0.258	0.041	.**009**	.**018**	.**004**	.**007 (more complex model)**
14	w_MW at wave (T2) → w_MW at wave (T3)	0.064	0.027	.**046**	.064		
15	w_CP at wave (T1) → w_CP at wave (T2)	0.132	0.035	.**003**	.241		
16	w_CP at wave (T2) → w_CP at wave (T3)	0.039	0.035	.**001**	.051		
17	w_SIF at wave (T1) → w_SIF at wave (T2)	0.138	0.027	.**020**	.**028**	.**009**	.**023** (more complex model)
18	w_SIF at wave (T2) → w_SIF at wave (T3)	0.258	0.041	.**009**	.176		
Between‐person random intercept effects							
19	RI_MW ~ RI_CP	1.618	0.205	.**016**	.108		
20	RI_MW ~ RI_ SIF	1.537	0.754	**<.001**	.**001**	**<.001**	.418 (quadratic)
21	RI_CP ~ RI_SIF	0.777	0.214	**<.001**	**<.001**	.880 (linear)	

*Note*: The bolded values indicate statistically significant results.

Abbreviations: CP, communication with parents; MW, mental well‐being; RI, random intercept; SIF, social interactions with friends; w, within‐person.

For the autoregression effects, significant age‐related variations were found in mental well‐being and social interaction with friends from Wave 1 to Wave 2 (*SD*_*p*s < .05; Table 5, Nos. 13 and 17). Further model fit analyses indicated that both the linear and quadratic models (*p*_fit_Linear vs. Local_ = .004, *p*_fit_Quadratic vs. Local_ = .007; see No. 13 in Table 5) were inadequate, suggesting the need for a more complex model (the plot is shown in Figure 2a). Similarly, for the autoregression effect of social interaction with friends from Wave 1 to Wave 2, both the linear and quadratic models (*p*_fit_Linear vs. Local_ = .028, *p*_fit_Quadratic vs. Local_ = .009; see No. 17 in Table 5) were inadequate, suggesting the need for a more complex model (the plot is shown in Figure 2b).

Regarding between‐person effects, age significantly moderated two random intercept correlations: (1) between mental well‐being and social interaction with friends, and (2) between frequency of communication with parents and social interaction with friends (*SD_ps* < .05), indicating significant variations in the standardized coefficient across age (Table 5; Nos. 20 and 21). Specifically, the relationship between mental well‐being and social interaction with friends showed a quadratic trend (*p*_fit_Quadratic vs. Local_ = .418, see No. 20 in Table 5), indicating a U‐shaped trend in the relationship (the plot is shown in Figure 2c). As for relationships between random intercepts of frequency of communication with parents and social interaction with friends, the test of sufficient fit showed a linear relationship with age (*p_fit*_Linear vs Local_ = .880, see No. 21 in Table 5). When considered in conjunction with the plot (Figure 2d), this supports the hypothesized linear moderation effect. The regression coefficients of the moderating effects of age on two random intercept correlations are shown in Table 6.

**TABLE 6:** Test of the moderator's linear and non‐linear regression coefficients.

Effects		coef	est	se	*t*	*p*
Linear						
1	RI_ communication with parents ~ RI_ social interactions with friends	(Intercept)	−0.437	0.308	−1.416	.157
		*m*	**0.090**	0.023	3.924	.000
Quadratic						
2	RI_Mental well‐being ~ RI_ social interactions with friends	(Intercept)	**23.717**	4.530	5.236	<.001
		*m*	**−3.559**	0.687	−5.180	<.001
		*I*(m^2^)	**0.138**	0.256	5.418	<.001

*Note*: The bolded values indicate statistically significant results.

Abbreviations: chisq_fit, the test of a sufficient fit of a parameter curve; chisq_het, the global test for parameter heterogeneity; coef, coefficients for the pre‐specifying moderation effects; est, the estimated value of coefficients; *p*, the *p*‐value of coefficients (calculated by t‐test); *p*_fit, the *p*‐value of the test of a sufficient fit of a parameter curve; *p*_het, the *p*‐value of the global test for parameter heterogeneity; RI, random intercept; se, the sample estimated standard error of coefficients; *t*, the estimated *t*‐value of coefficients.

## DISCUSSION

Extant studies have highlighted the importance of both parents and peers for adolescent well‐being, yet the findings remain inconsistent. Although interactions with both can complement each other to enhance adjustment, evidence regarding their relative importance has been mixed (Oldfield et al., 2016). Moreover, the bi‐directional relationships between adolescents and their social contexts—where adolescents' development both predicts and is predicted by interaction with parents and peers—remain underexplored. To address these gaps, the current study integrates latent structural equation modeling (LSEM) into RI‐CLPM to simultaneously examine within‐person and between‐person effects between parent/peer interactions and adolescent mental health and to explore potential age‐related moderation.

### Relationships between mental well‐being and social interactions: Cross‐lagged effects

For within‐person effects, our results suggest significant cross‐lagged effects in adolescent mental well‐being and parent/peer interactions, although these findings only partially supported our hypotheses. First, the significant cross‐lagged effect of the frequency of communication with parents on mental well‐being suggests that higher levels of parent–adolescent communication predict higher levels of mental well‐being over time. Moreover, the cross‐lagged effects of the frequency of communication with parents on mental well‐being exhibited no significant variation across different age groups, underscoring the consistent role of parent–adolescent communication in predicting adolescent mental well‐being from ages 10 to 18 years. This finding aligns with previous research, emphasizing the pivotal role of parent–child communication in predicting adolescent well‐being (Kerr & Stattin, 2000). The possible reasons may be that, through parent‐driven communication (e.g., monitoring and behavioral control) or adolescent‐driven communication (e.g., voluntary disclosure; Ding et al., 2022; Kapetanovic & Boson, 2022), parents gain awareness of adolescents' daily activities, which may enable them to implement strategies that support growth and protect against risks (Kerr et al., 2010; Kerr & Stattin, 2000). Additionally, adolescents' openness to parents may foster a sense of connectedness and belonging and contribute to affective qualities of parent–child relationships, which in turn may support their mental health (Keijsers & Poulin, 2013).

However, the reverse cross‐lagged effects (mental well‐being predicting future frequency of communication with parents) were not significant, indicating that although parental communication predicts adolescent mental well‐being, mental health does not predict more frequent communication with parents, and age did not moderate this relationship. The finding highlights the asymmetrical nature of parent–adolescent dynamics, showing that parent–adolescent interactions predict adolescent outcomes over time, whereas adolescent outcomes show no significant predictive association with subsequent parent–child interactions. This pattern aligns with previous findings. For instance, prior studies have shown that maternal use of expressive suppression relates to adolescents' higher depressive symptoms, but adolescents' use of expressive suppression did not relate to maternal depression (Su et al., 2024; Wolff et al., 2020). The underlying reason for this imbalance can be attributed to the developmental and hierarchical nature of the parent–child interactions. It is important to mention that many contemporary parenting theories posit that parents and children mutually affect one another, especially during adolescence, when children become more active agents within the parent–child relationships (Pardini, 2008). Our results, which suggest asymmetrical parent–adolescent processes in specific domains of communication and mental well‐being, do not contradict these theories, given that parent–child reciprocity is not universal but varies depending on assessed variables, parenting behaviors, and individual factors. For example, environmental sensitivity and neuroticism appear to be promising traits for understanding why some adolescents are more strongly affected by parenting than are others (Boele et al., 2023). Future studies should further examine the conditions under which parent–adolescent reciprocity emerges by considering a broader range of adolescent outcome domains, parenting behaviors, and individual characteristics.

Second, cross‐lagged effects suggest that higher levels of adolescent mental well‐being are associated with subsequent increases in social interactions with friends, rather than the reverse. These results highlight the potential role of mental well‐being as a preceding factor, rather than as an outcome, in relation to social interactions. According to Fredrickson's broaden‐and‐build theory (Fredrickson, 2013), positive affect broadens adolescents' thought‐action repertoires, increasing motivation and capacity for social engagement. Thus, adolescents with higher mental well‐being may be more likely to initiate and maintain peer interactions. Our finding contrasts with previous findings that peer interactions predicted adolescent adjustment (Schwartz‐Mette et al., 2020; Widnall et al., 2022). The use of RI‐CLPM in this research, unlike in previous research, may help explain this discrepancy, as it allows for a more precise understanding of how within‐individual changes in mental well‐being influence social behavior, which may not be captured in traditional models that focus on overall group‐level effects. Another possible explanation for why social interactions with friends did not predict adolescent mental health is that our measure captures the frequency of peer interactions rather than their quality or supportive nature. Theoretically, an increase in frequency does not necessarily provide emotional support, and it is often high‐quality social interactions that are most likely to enhance mental well‐being. For example, using retrospective data, one study has found that adolescent peer support quality, rather than the number of friends, uniquely predicts depressive symptoms in adolescence and adulthood (Letkiewicz et al., 2023).

In addition, the cross‐lagged effects revealed asymmetrical and distinct patterns for parents and peers, with parent interactions predicting adolescent well‐being over time (but not vice versa) and adolescent well‐being predicting peer interactions (but not vice versa). Unexpectedly, LSEM analysis found no significant moderation by age, challenging the view that peers become more important and parents become less important as adolescents grow (Keijsers & Poulin, 2013; Lam et al., 2014). These findings suggest that, in Chinese culture that emphasizes family relatedness and parental authority, parent–adolescent interaction may serve as a stable and enduring source of support, consistently promoting adolescents' mental well‐being across development and exerting a stronger influence than friendships. Consistent with these results, a recent cross‐sectional study of Chinese adolescents (*M* = 15.89 years, *SD* = 0.81) identified four attachment profiles to parents and peers: high insecurity to both, secure parents but insecure peers, insecure parents but secure peers, and high security to both (He et al., 2018). They found that adolescents with high insecurity to both parents and peers showed the lowest well‐being and highest distress, whereas those with high security to both showed the highest well‐being and lowest distress (He et al., 2018). However, adolescents with insecure parents but secure peers had more emotional distress than adolescents with secure parents but insecure peers, indicating that insecure parent attachment may contribute more strongly to psychological distress than insecure peer attachment in middle adolescence (He et al., 2018).

### Relationships between mental well‐being and social interactions: Between‐person effects

Between‐person effects help identify individuals who are at consistent risk due to stable, long‐term patterns of behavior or interaction, allowing targeted interventions for consistently disadvantaged groups. Our findings suggest that adolescents who, on average, have higher levels of mental well‐being tend to engage more frequently in conversations with their parents and social interactions with their peers. These results are consistent with past research linking supportive parent and peer interactions to adolescent well‐being (Kerr & Stattin, 2000; Nowell et al., 2023; Stattin & Kerr, 2000).

Age was found to moderate the random intercept correlations between mental well‐being and social interactions with friends, showing a U‐shaped trend in the relationship. The association was weakest around ages 13 and 14 years, corresponding to the first year of middle school.

This pattern may be explained, in part, by evidence that early adolescents, compared with both children and adults, exhibit heightened sensitivity to peer evaluations and increased reward sensitivity in social contexts (Giletta et al., 2021). Past literature has also shown that for both girls and boys, unsupervised time with same‐sex peers increased from age 8 years to about age 14 years and then declined (Lam et al., 2014), suggesting that around age 14 years, there may be a developmental shift in peer interactions. Nevertheless, the study by Lam et al. (2014) observed a slight cubic trend between adolescent peer interaction and adolescent depression, suggesting that depressive symptoms may fluctuate nonlinearly with age; however, no information was provided regarding the specific turning points of this trend. More research is needed to clarify the developmental timing and mechanisms underlying these nonlinear associations. Another possible explanation for the U‐shaped trend is that at around 13 to 14 years old, adolescents undergo a major transition as they graduate from primary school and enter middle school. This phase, marked by adapting to new peer groups and unfamiliar school dynamics, likely weakens the association between mental well‐being and social interactions. By ages 16 and 17 years, when students enter high school, this association tends to strengthen even further. This is likely because high school students in China often live on campus, leading to closer peer relationships through increased daily interactions and shared experiences.

In addition, age moderated the between‐person association between parent–adolescent communication and mental well‐being. Specifically, older adolescents showed a stronger positive association between communication with parents and mental well‐being than younger adolescents. Similar findings have been reported in previous studies. For example, using a cross‐sectional design, one study with Chinese adolescents has shown that the negative associations that both father–adolescent and mother–adolescent communication had with adolescent depressive symptoms were stronger in ninth‐grade students than in seventh‐grade students (Zhang et al., 2021). One possible interpretation is that older adolescents, who typically face more diverse academic demands, social expectations, and autonomy‐related challenges than younger adolescents, may differ more markedly in how they draw on parental communication as a source of emotional support, guidance, or validation. Under these age‐related conditions, variations in the quality of parent–adolescent communication may be more consistently reflected in between‐person differences in mental well‐being.

### Stability of mental well‐being and social interactions

As expected, the within‐person autoregressive effects for all three variables—mental well‐being, frequency of communication with parents, and social interactions with friends—were significant across consecutive time points, suggesting a high degree of stability in these behaviors and emotional states. However, previous findings regarding the stability of social interactions and adolescent well‐being have been inconsistent. Meuleman et al. (2024) have found stable caregiver support but unstable adolescent symptom distress across three 6‐month intervals. A critical factor that may explain these discrepancies is the measurement interval length. Our research used a shorter interval of 3 months, which may not capture fluctuations because these variables appear to be relatively stable over shorter periods. Future research should consider using a combination of shorter and longer intervals to better understand the temporal dynamics of these variables.

Moreover, the LSEM results showed that age moderated the autoregressive effects of adolescent mental well‐being and social interaction with friends, suggesting that their stability varies with age. Rather than following a simple linear or quadratic trend, these age‐related changes appear to follow a more complex, higher‐order pattern. This means that the stability of mental well‐being and social interactions with friends across different age groups may be influenced by additional factors, and the pattern of change could be more intricate. Past research has reported developmental changes in parent–child communication from early to late adolescence. For example, one study has examined how parent–child communication regarding adolescent unsupervised activities develops over the course of adolescence from age 12 to 19 years (Keijsers & Poulin, 2013). They found that boys' disclosure decreased in early adolescence (age 12 to 14 years) and was stable thereafter; for girls, however, while disclosure also decreased between ages 12 and 14 years, there was an increase in disclosure in middle adolescence (Keijsers & Poulin, 2013).

### Relationships between interactions with parents and interactions with peers

Although not the major focus of this research, the results showed no significant cross‐lagged effect between social interactions with friends and the frequency of communication with parents, indicating that short‐term changes in one type of interaction do not necessarily predict immediate changes in the other. However, a significant correlation was observed between the random intercepts of these variables, indicating that adolescents who engage in more frequent conversations with their parents tend to have higher levels of social interactions with friends. The relationship between these random intercepts showed a significant linear trend with age, suggesting that the association between parent and peer interactions strengthens as adolescents develop. A possible explanation is that both types of interactions rely on similar core social skills, such as empathy, active listening, and effective communication. Socially skilled adolescents are likely to apply these abilities across different contexts, leading to frequent interactions with both parents and peers. Consistent with our findings, one study has found that adolescents who reported more computer‐mediated communication time with friends tended to have more face‐to‐face time with parents (Manago et al., 2020).

### Theoretical and practical implications

Relative to other periods of the life course, there has been surprisingly little research that takes account of the dynamism of human interactions to investigate how adolescent relationships may contribute to mental health (Pachucki et al., 2015). As adolescents become increasingly autonomous, they begin to transfer their dependencies from their parents onto their peers. These different social interactions play complementary roles in shaping adolescents' psychological adjustment, highlighting the importance of considering both parental and peer influences in developmental research. Nevertheless, considerable debate continues over whether parents or peers are more strongly associated with adolescent well‐being (Oldfield et al., 2016). Indeed, only a handful of studies have included different types of relationships within the same piece of research with a mental health construct as the outcome (Oldfield et al., 2016). This study provides new insights into this debate by simultaneously examining parent–adolescent and peer interactions across a wide developmental period in China. By employing longitudinal designs that disentangle between‐person from within‐person processes, the study provides evidence that parent–adolescent interaction exerts a stronger predictive role on adolescent well‐being compared with peer interactions, regardless of age.

As noted by past literature, traditional Chinese culture emphasizes the interests of the collective over those of the individual, and within the family, individuals are expected to respect authority, particularly that of parents (Wenxin et al., 2006). Despite rapid social and economic changes, these values remain influential. Compared with Western peers, Chinese adolescents tend to accept parental authority more and seek independence later (Wenxin et al., 2006). Thus, although this study highlights the stronger predictive role of parent–adolescent interaction in Chinese adolescents' well‐being, cultural norms around family obligation, parental authority, and independence may moderate these dynamics across cultures. In Western contexts, where autonomy and peer affiliation are emphasized, the relatively important roles of peer versus parent relationships are warranted to be further examined. Cross‐cultural comparative studies are needed to determine whether the patterns observed here reflect universal developmental processes or culture‐specific pathways. Nevertheless, the findings of this study suggest that interventions aimed at enhancing family communication may yield substantial benefits for Chinese adolescents' psychological well‐being, irrespective of their developmental stage.

Parents are responsible for guiding their children and supporting positive development during adolescence. A key strategy that enables such guidance and protection is maintaining knowledge of adolescents' daily lives. When parents are aware of adolescents' everyday activities, they are better positioned to apply effective parenting practices that support healthy development and safeguard adolescents from potential risks (Kapetanovic & Boson, 2022; Kerr & Stattin, 2000). This knowledge can be gained through parent‐driven communication, such as solicitation (actively monitoring and asking for information) and behavioral control (establishing behavioral rules), as well as through adolescent‐driven communication, namely, voluntary disclosure of activities (Kerr & Stattin, 2000; Stattin & Kerr, 2000). Our results, showing that parent–adolescent communication predicts adolescents' subsequent mental well‐being, may have roots in the role of communication as a means for parents to monitor, guide, and support their children's positive development. Another possible explanation for the positive predicting roles of parent–adolescent communication on adolescent mental well‐being is that such communication facilitates a sense of connectedness between parents and adolescents, which in turn can promote psychological well‐being. For example, prior research has documented that parent–child connectedness was associated with more body satisfaction for females, higher levels of self‐esteem for males, and fewer depressive symptoms for both males and females over 5 years in a diverse sample of adolescents (Boutelle et al., 2009).

In addition, at the between‐person level, the correlations between parent–adolescent communication, peer interaction, and adolescent well‐being were positive. Although these relationships are correlational and do not imply causation, they provide valuable information for identifying adolescents who may be at greater risk than their peers—specifically, those who exhibit problematic interactions with parents or peers. These findings highlight the importance of monitoring relational patterns across different social contexts, as difficulties in either family or peer relationships may serve as indicators of lower mental well‐being in situations where well‐being cannot be assessed directly.

### Limitations and future research directions

Despite the strengths, several limitations should be acknowledged. First, the data used in this research spanned three time points, each 3 months apart. Longer intervals between measurements, such as 6 months or more than 1 year, could offer better insights into the stability and long‐term effects of the relationships, capturing enduring changes and developmental trends. Second, the data were collected using self‐report measures, which may be subject to biases, such as social desirability or inaccurate recall. Future studies could incorporate multi‐informant reports (e.g., parents, peers, or teachers) or objective measures to reduce bias and provide a more well‐rounded view of adolescents' experiences. Third, although our sample was large and diverse, it was limited to Chinese adolescents. Cross‐cultural studies are needed to determine the generalizability of these findings to other cultural contexts, as adolescents in diverse cultures may experience different family dynamics, peer interactions, and socialization practices. Fourth, social interactions with friends and the frequency of communication with parents were each measured using only two items. Although these measures provide a basic understanding of social interaction, they may not capture the quality or depth of these interactions, which are important in understanding their effect on adolescent well‐being. Future research should incorporate comprehensive measures that assess the frequency, quality, and content of these interactions.

## CONCLUSIONS

This research provides valuable insights into the complex interplay between mental well‐being, parent–child communication, and peer interactions among Chinese adolescents using RI‐CLPM and LSEM. The findings show that the autoregressive effects of the three variables were significant, indicating a high degree of stability in these behaviors and emotional states. Higher levels of communication with parents predicted better mental well‐being, while better mental health predicted more peer interactions, revealing asymmetrical patterns of influence that remained stable across different ages. Adolescents with higher average levels of mental well‐being tended to engage more frequently in conversations with their parents; this association tended to be stronger with age increase. Similarly, those with higher levels of mental well‐being tended to engage more frequently in social interactions with friends, though this association followed a U‐shaped trend.

## CONFLICT OF INTEREST STATEMENT

The authors report no conflicts of interest.

## ETHICS STATEMENT

This study was approved by the Human Subjects Protection Ethical Review Committee of Tsinghua University. All procedures were conducted in accordance with the ethical standards of the Declaration of Helsinki.

## INFORMED CONSENT

Prior to participants' involvement, students' assent to participate and parental informed consent were obtained.

## DECLARATION OF GENERATIVE AI AND AI‐ASSISTED TECHNOLOGIES

During the preparation of this work, the authors used ChatGPT to improve phrasing and grammar. After using this tool, the authors reviewed and edited the content as needed and take full responsibility for the content of the publication.

## Supporting information


**Table S1** Comparison of demographic variables and major assessed variables between adolescents who participated in Wave 1 assessment, and those who participated in all three assessments.
**Table S2** Within‐Person Cross‐Lagged Effects in the Sensitivity Analysis Using the Complete Sample.
**Figure S1** Histogram of age.

## Data Availability

This study was not preregistered. The datasets, study materials, and analysis code are available from the corresponding author on reasonable request.
