# Supplementary material for: Who plays a more crucial role in adolescent well‐being: Interactions with parents or peers? An investigation of adolescents aged 10 to 18 years
Source: Appl Psychol Health Well Being. 2026 Mar 6;18(2):e70128. doi: 10.1111/aphw.70128 (PMC12965047; doi:10.1111/aphw.70128)

**Supplementary Materials**

Table S1 Comparison of demographic variables and major assessed variables between adolescents who participated in Wave 1 assessment, and those who participated in all three assessments.

Table S2 Within-Person Cross-Lagged Effects in the Sensitivity Analysis Using the Complete Sample

Figure S1 Histogram of age

**Table S1**

*Comparison of demographic variables and major assessed variables between adolescents who participated in Wave 1assessment, and those who participated in all three assessments.*

|  |  | Wave 1  (n= 35,079) | All three waves  (n= 33,824) |
| --- | --- | --- | --- |
| Region | *Urban area* | 27,604 (78.69%) | 26,361 (77.94%) |
|  | *Rural area* | 7,475 (21.31%) | 7,463 (22.06%) |
| School | *Public school* | 28,085 (80.06%) | 26,854 (79.39%) |
|  | *Private school* | 6,994 (19.94%) | 6,970 (20.61%) |
| Gender | *Male* | 18,512 (52.77%) | 17,782 (52.57%) |
|  | *Female* | 16,567 (47.23%) | 16,042 (47.43%) |
| Age | *Mean* | 13.28 | 13.33 |
|  | *SD* | 2.23 | 2.24 |

**Table S2**

*Within-Person Cross-Lagged Effects in the Sensitivity Analysis Using the Complete Sample*

| Estimation | | T1 ‎→ T2 | | T2 ‎→ T3 | |
| --- | --- | --- | --- | --- | --- |
|  |  | $\beta$ | $p$ | $\beta$ | $p$ |
| Within-Person Autoregressions | | | | | |
|  | Mental Well-Being → Mental Well-Being | **.101 [.082, .121]** | **<.001** | **.246 [.229, .263]** | **<.001** |
|  | Communication with Parents →Communication with Parents | **.075 [.056, .093]** | **<.001** | **.147 [.129, .164]** | **<.001** |
|  | Social Interactions with Friends → Social Interactions with Friends | **.046 [.028, .064]** | **<.001** | **.131 [.114, .148]** | **<.001** |
| Within-Person Cross-Lagged Effects | | | | | |
|  | Communication with Parents → Mental Well-being | **.033 [.021, .045]** | <.001 | **.033 [.021, .045]** | <.001 |
|  | Social Interactions with Friends → Mental Well-being | -.007 [-.017, .004] | 0.221 | -.007 [-.017, .004] | 0.221 |
|  | Mental Well-being →Communication with Parents | **.017 [.006, .030]** | 0.007 | **.019 [.006, .034]** | 0.007 |
|  | Mental Well-being → Social Interactions with Friends | **.023 [.011, .036]** | <.001 | **.025 [.012, .039]** | <.001 |
|  | Communication with Parents → Social Interactions with Friends | -.003 [-.015, .009] | 0.674 | -.003 [-.015, .009] | 0.674 |
|  | Social Interactions with Friends →Communication with Parents | .004 [-.007, .015] | 0.511 | .004 [-.007, .015] | 0.511 |

**Figure S1**

*Histogram of age*


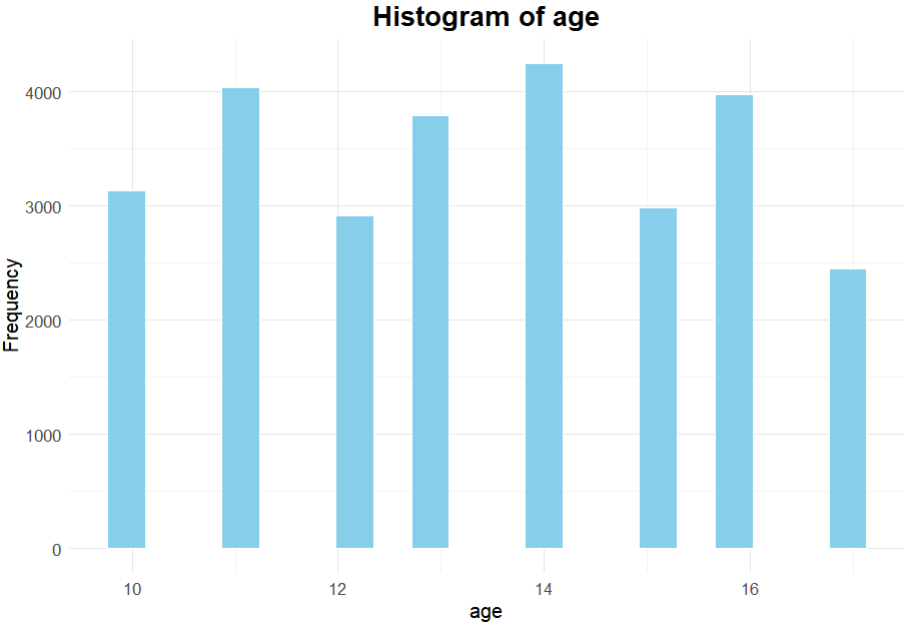

Supplement: Supplementary file 1 — Table S1 Comparison of demographic variables and major assessed variables between adolescents who participated in Wave 1 assessment, and those who participated in all three assessments. Table S2 Within‐Person Cross‐Lagged Effects in the Sensitivity Analysis Using the Complete Sample. Figure S1 Histogram of age. [file APHW-18-0-s001.docx]
